# COVID-19 activity risk calculator as a gamified public health intervention tool

**DOI:** 10.1038/s41598-023-40338-8

**Published:** 2023-08-11

**Authors:** Shreyasvi Natraj, Malhar Bhide, Nathan Yap, Meng Liu, Agrima Seth, Jonathan Berman, Christin Glorioso

**Affiliations:** 1 Department of Neurosciences, Faculty of Medicine, University of Geneva, Geneva, Switzerland; 2 Department of Anatomy, University of California, San Francisco, CA USA; 3 Academics for the Future of Science Inc., Cambridge, MA USA; 4 Department of Industrial and Manufacturing Engineering, Penn State University, State College, PA USA; 5 School of Information, University of Michigan, Ann Arbor, MI USA; 6 Department of Basic Science, New York Institute of Technology College of Osteopathic Medicine at Arkansas State University, Jonesboro, AR USA

**Keywords:** Risk factors, Epidemiology, Population screening

## Abstract

The Coronavirus disease 2019 (COVID-19) pandemic, caused by the virus severe acute respiratory syndrome coronavirus 2 (SARS-CoV-2), has impacted over 200 countries leading to hospitalizations and deaths of millions of people. Public health interventions, such as risk estimators, can reduce the spread of pandemics and epidemics through influencing behavior, which impacts risk of exposure and infection. Current publicly available COVID-19 risk estimation tools have had variable effectiveness during the pandemic due to their dependency on rapidly evolving factors such as community transmission levels and variants. There has also been confusion surrounding certain personal protective strategies such as risk reduction by mask-wearing and vaccination. In order to create a simple easy-to-use tool for estimating different individual risks associated with carrying out daily-life activity, we developed COVID-19 Activity Risk Calculator (CovARC). CovARC is a gamified public health intervention as users can ”play with” how different risks associated with COVID-19 can change depending on several different factors when carrying out routine daily activities. Empowering the public to make informed, data-driven decisions about safely engaging in activities may help to reduce COVID-19 levels in the community. In this study, we demonstrate a streamlined, scalable and accurate COVID-19 risk calculation system. Our study also demonstrates the quantitative impact of vaccination and mask-wearing during periods of high case counts. Validation of this impact could inform and support policy decisions regarding case thresholds for mask mandates, and other public health interventions.

## Introduction

The risk levels for gatherings and activities during the COVID-19 pandemic have fluctuated significantly since its onset^1,2^. These fluctuations have been driven by variation in the determinants of infection, hospitalization, and death risk, which include community transmission levels, size of gatherings, social distancing, vaccination status, type of vaccine, the dose of vaccine, face mask usage, air filtration, circulating SARS-CoV-2 variants, health conditions, age, gender, previous infection, and time since previous infection or vaccination (due to waning immunity)^3^. The risk of becoming infected with SARS-CoV-2 in the environments most conducive to spread (indoor and crowded) are significantly higher at peak community case levels than at lower community case levels^4–6^. Due to the rapidly evolving nature of these risk levels and the number of determinants involved^7^, it has been challenging for the public to maintain a clear understanding of what their risk is for various activities over time. As a result, some non-scientist citizens have created tools^8^ to try to assess risk for their households, and publications such as the New York Times^9^ have attempted to create risk assessment algorithms. However, these manual attempts can be complicated to use and lacking in enough determinants to accurately estimate risk, resulting in confusion over cost-benefit surrounding harm reduction strategies such as the use of face masks, restrictions on gathering size, and vaccination, creating an ”infodemic”^10^.

Research teams have created various tools for individuals to evaluate their risk^11^ (see Table 1), each taking a somewhat different approach. A simple approach taken by many Dashboards, including the US Center for Disease Control (CDC) is to report risk levels on a county-wide basis simply by community transmission levels^12^. While this is a simple to understand and a useful metric, it doesn’t address differences in risk by individual risk factors such as age and health condition, differences in activities such as number of attendees, or differences in precautions such as mask-wearing or vaccination. All of these factors are important and can change risk drastically.

**Table 1:** This table provides a comparison between different risk calculators on the basis of the time each user needs in order to carry out the risk estimation process (Fast or not), the factors that each of the risk calculator considers when carrying out risk calculation (whether it considers vaccination, usage of mask, past health conditions, presence of COVID-19 variants, number of people the user is going to be in contact with and if the specific activity the user is going to be performing is indoors or outdoors). We also include number of locations around the world that the calculator works for and if they take into account specific activities (such as going to supermarket, football practice etc.). We then check if there the currently existing risk calculators estimate overall risk or also provide risk of infection, hospitalization and death separetely. We then identify the drawbacks and areas where the currently existing risk calculators are lacking in order to create our risk calculator which is fast, takes into account a majority of factors when estimating risk and estimates different levels of risk (infection, hospitalization and death).

Risk calculator	Is	Considers								Calculates the risk of		
	Fast	Vaccination	Health conditions	Many locations	Mask type	Number of people	Indoor or outdoor	Variants	Specific activities	Infection	Hospitalization	Death
CovARC (Our calculator)	X	X	X	X	X	X	X	X		X	X	X
QCovid^13^	X		X								X	
ASIMI model^14^	X				X		X			X		
19 an Me (Princeton model)^15^		X	X						X	X		
MyCOVIDRisk^16^	X				X	X	X		X	X		
COVID-19 Assessment tool (GA Tech Model)^17^	X			X						X		
Covidtracker.fr^18^	X									X		
Max Planck Institute COVID risk calculator^19^					X		X	X		X		
COVID-19 Indoor safety Guide (MIT Model)^20^	X				X		X	X		X		
COVID-19 Risk calculator (Northwestern University Model)^21^	X		X									X
microCOVID Project^8^		X		X	X	X	X			X		

The approach factoring in the smallest number of determinants is the COVID-19 Event Risk Assessment Planning Tool, a web-based tool^17^, developed by scientists at the Georgia Institute of Technology. The tool estimates the probability that a person will encounter someone infected with SARS-CoV-2 at a particular gathering based on the group’s size and the event’s location (factoring in under-reporting adjusted community transmission levels). The tool works for the US only, at the county level and does not consider the individual’s health risk factors, vaccination status, variants, mask usage, or indoor v. outdoor activities. It estimates the chances that someone at a gathering of a user-set size will be actively infected with SARS-CoV-2.

The 19 and Me calculator^15^, developed by Mathematica, a policy-research company in Princeton, New Jersey, draws on demographic and health information and user behaviors such as hand washing and masks used to determine the relative risk of exposure, infection, and severe illness for the individual’s behavior every week. It does not account for variants and only works for the US and Belgium.

In December 2020, a team led by biostatistician Nilanjan Chatterjee at Johns Hopkins University in Baltimore, Maryland, released the COVID-19 Mortality Risk Calculator^22^, which estimates an individual’s relative risk of death from COVID-19 during an activity based on their location, pre-existing conditions, and general health status. It also reports the estimated risk of death in a user’s area in the next two weeks. Activities, personal precautions, vaccination, and variants are not factored in.

Another approach for risk calculator called MyCOVIDRisk^16^ takes a more situational approach, estimating the risks associated with specific errands or recreational activities. The estimate is based on the location, duration, and the number of masked or unmasked people attending. This can help users avoid activities that are likely to be high risk in a specific pandemic hotspot, such as spending an hour or more at an indoor gym, favoring safer alternatives—a masked meet-up in the park, for instance,^23^. It does not report risk numerically, instead opting for a low-very large scale, which does not consider an individual’s risk tolerance threshold. It also does not account for variants.

We build upon the comprehensiveness of these tools with more factors taken into account and more geographical locations. To our knowledge, this estimation method is the only one that considers variants and vaccine coverage and works in almost every country. The app is lightweight and can be used on low bandwidth internet, which is an important factor in many countries. We aim to aid people worldwide in making informed decisions about how to do activities more safely during a global Pandemic.

## Results

**Figure 1:**
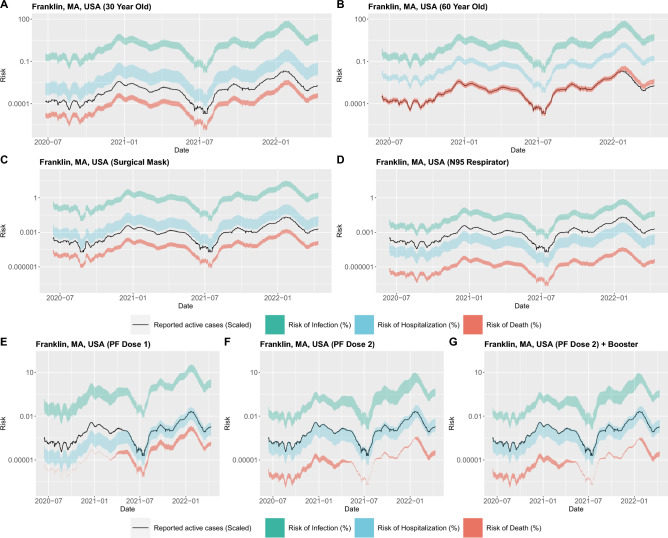
In the following figures, we use the location of Franklin, Massachusetts, United States of America and calculated the range of risks of infection, hospitalization and death for a (textbfA)30-year-old male with no chronic illness, no mask and no vaccination, 10 people passed outdoors and 5 people passed indoors during the activity, a (textbfB)60-year-old male with no chronic illness, no mask and no vaccination, 10 people passed outdoors and 5 people passed indoors during the activity. A (textbfC)30-year-old male with no chronic illness, surgical mask and no vaccination when 10 people are passed outdoors and 5 people passed indoors during the activity, a (textbfD)30-year-old male with no chronic illness, N95 respirator mask and no vaccination when 10 people are passed outdoors and 5 people passed indoors during the activity, a (textbfE) 0-year-old male with no chronic illness, no mask and Dose 1 of Pfizer vaccination when 10 people are passed outdoors and 5 people indoors during the activity, a (textbfF)30-year-old male with no chronic illness, no mask and Dose 2 of Pfizer vaccination when 10 people are passed outdoors and 5 people indoors during the activity and a (textbfG)30-year-old male with no chronic illness, no mask and Dose 2 with a booster dose of Pfizer vaccination when 10 people are passed outdoors and 5 people indoors during the activity.

We implement here a simple COVID-19 Activity Risk Calculator (CovARC). We extract the number of confirmed cases by using the Johns Hopkins dataset^24,25^ and cross-validate the number of confirmed cases using the Facebook surveys^26^ dataset in order to obtain the upper and lower limits of the number of confirmed cases. We subtract confirmed cases from the preceding day’s number of confirmed cases in order to identify the number of active cases. These active cases are then taken into account to create a 14-days aggregate. We then divide the 14-days aggregate by the population of the city/state in order to identify the density of COVID-19 cases. We use this as the actual density of active cases which is then taken by the risk calculator in order to estimate the risk. Apart from the number of active cases, we take into account the presence of alpha, beta, gamma, delta and omicron variants using the variants dataset^27^ to identify the influence of variants on the risk of infection. The variants data is coupled with the vaccine efficacy data (see Table 2 B) in order to obtain the final influence of variants and vaccines on the overall simulated range of risk of infection (see Fig. 1D–F). We also took into account the influence of mask wearing (see Table 2 A) on the overall simulated range of risk of infection from COVID-19 (see (Fig. 1C,D). Since there are several types of risk associated with COVID-19 (infection, hospitalization, and death), we included inputs that change these risks differentially, including COVID-19 variants, and health and demographic factors of the user including, age, sex, and chronic illness (see Table 3) in order to obtain the simulated range of risks of hospitalization and death for different scenarios (see Fig. 1A,B).

Using several different datasets and references we obtained the results using our risk calculation system over time showcasing the first outbreak of COVID-19 followed by alpha, beta, gamma, and delta variants outbreak and the omicron variant outbreak. Using our risk calculator, we demonstrate that the risk level increases with a high number of active cases, older age, less vaccination, and lower quality or no face mask usage (see Fig. 1). We also observed that the risk also increases when carrying out indoor activities, more density of COVID-19 cases, vaccine type, previous chronic illnesses, male sex, and presence of variants of concern (see Table 3).

**Figure 2:**
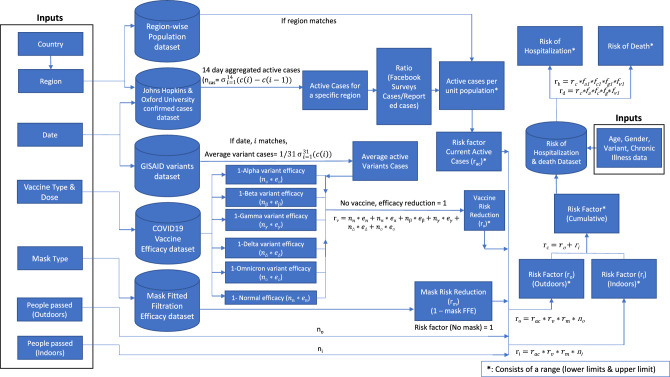
This figure represents a simplified workflow diagram of the risk calculation along with different datasets as well as other inputs used in order to estimate different risks related to COVID-19. The diagram also represents the steps that are performed when calculating the risk reduction when different preventive measures are taken.

**Figure 3:**
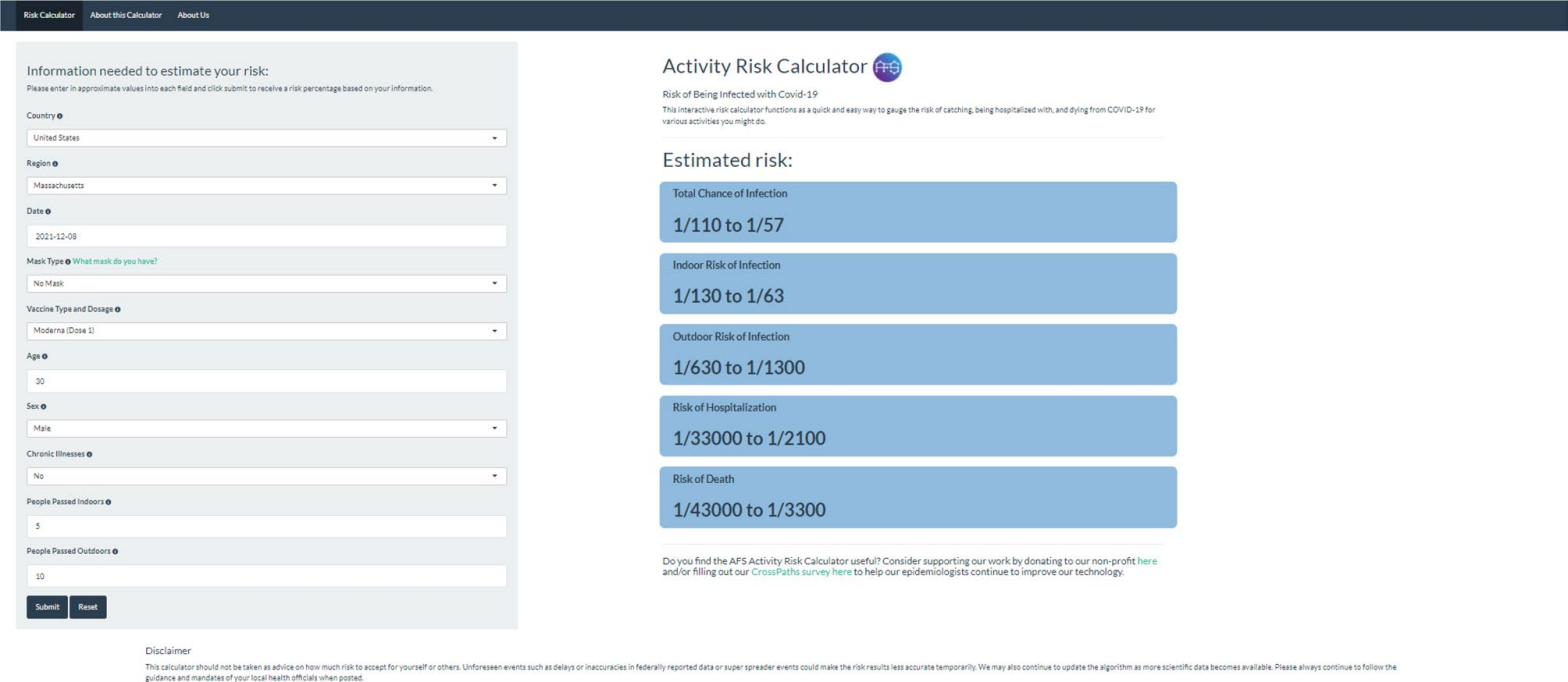
The given image is a screenshot of the user interface of the web application that was translated using the risk calculation system. The web application uses a form-like structure with minimal fields to ensure that users can carry out risk estimation as quickly as possible. After clicking the submit button, the results are displayed on the right partition of the web application. Using this, the user can identify different personal risks for carrying out a specific daily life activity.

We additionally implemented the code in the form of a streamlined web application that inputs user supplied variables and generates their simulated risk scores (see Fig. 3) to create a tool that can be used by anyone anywhere in the world to get a risk score related to COVID-19 when they are carrying out a daily life activity. A simplified flow diagram that outlines the risk calculator’s algorithm can be found in Fig. 2.

To illustrate how risk changes temporally and with different variables, we looped over time from June 2020 until April 2022 under various conditions and plotted the results. The different test scenarios that were performed can be seen as follows.

### Change in risk of infection, hospitalization and death with community transmission levels and age

In Fig. 1A, we first take the scenario of a 30-year-old male with no chronic health conditions located in Franklin, Massachusetts, USA. We set the conditions to be that the individual is not vaccinated and is not wearing a mask. We set the activity of this person to involve close contact (within 6 feet) with five people indoors and ten people outdoors. We then observe the simulated range of risks of infection, hospitalization, and death over time. There was a close correlation between the change in the number of active cases and the simulated range of risks of infection, hospitalization, and death.

The three peaks in the plot highlight the three COVID-19 outbreaks^28^ that took place in the world, the original outbreak, the outbreak of the alpha, beta, gamma, and delta variants, as well as the third outbreak of omicron. We then used the same inputs but altered the age of the individual to 70 years old and observed the same simulated range of risk of infection but a much-elevated simulated range of risk of hospitalization and death (see Fig. 1B) indicating that the range of risks of hospitalization and death are higher for an older individual compared to an individual who is younger.

### Change in risk of infection with vaccination and dosage

We explored the extent that vaccination and boosting with various vaccines decreases the risk of COVID-19. In order to do so we took the same scenario of a 30-year-old male with no chronic illness located in Franklin, Massachusetts, USA who is not vaccinated and compared it to if he had the first dose of the Pfizer Vaccine, the second dose of Pfizer Vaccine, and the second dose of the Pfizer Vaccine plus the Moderna/Pfizer Booster Vaccine. Using these three scenarios, we again calculated simulated risk for dates spanning the length of the COVID-19 Pandemic and obtained the results (see Fig. 1E–G).

We observed a reduced risk of infection upon vaccination with the first dose of the Pfizer vaccine and further risk reduction with dose 2 and booster dosage. We also observed that the risk of infection with omicron variants (observed from the third peak) is not significantly decreased by the dosage of the vaccination compared to other variants (observed in the second peak) indicating that precautions are still needed to be followed to ensure that there is less chances of infection from omicron variant.

### Change in risk of infection with mask type

We examined risk stratification by the usage of face masks. We experimented by taking 3 different scenarios using the first persona of an unvaccinated 30-year-old male with no past chronic illness in Franklin, Massachusetts, USA. We compared the original scenario where the individual was not wearing a mask to two other scenarios where he is wearing a 2-layer woven nylon mask with a nose bridge and one where he is wearing an N95 respirator^29^.

We observed that there is a significant reduction in risk of infection upon wearing a nylon mask with a nose bridge and a very high reduction in risk of infection when wearing the N95 respiration which was in line with several past studies conducted^30^. Moreover, depending upon the fitted filtration efficacy of the mask, there was a significant reduction in the risk of COVID-19 for both outdoor and indoor activities (Fig. 1C,D). This study also helped us to understand the extent of the decrease in risk of COVID-19 by just usage of masks and indicated that masks played a more important role compared to vaccination in the prevention of risk of COVID-19 when there is an outbreak.

We further carried out another secondary test by taking the location of Delhi, India to estimate different risks associated with COVID-19 as well as a reduction in risk due to different dosages of vaccination and usage of masks to check the robustness of our risk calculator for different locations across the world (see Supplementary Fig. S1 A–F).

### Ease of usage and streamlined estimation of COVID-19 risk

After carrying out these studies, we proceeded to make a user interface for CovARC so that it could be implemented as a usable tool by the general public^31^. Using RShiny^32^, we deployed a web application that could be used as a way for any user to estimate different risks with carrying out a specific daily life activity by providing input of their specific case.

By identifying the significant inputs required for calculating several risk factors pertaining to COVID-19 and minimizing the user inputs, we streamlined the risk calculation process at low bandwidths (see Fig. 3). Our goal was to implement our study as a tool that could be used on a day-to-day basis. Furthermore, the tool could also enable the user to get acquainted with the range of risks of infection, hospitalization, and death and identify ways in which the risks could be reduced.

## Discussion

We demonstrate that CovARC is an accurate and valuable tool for the public and policymakers. The risk estimation system is more comprehensive and simpler than existing alternatives. Due to its streamlined interface, the user is enabled to get acquainted with the range of risks of infection, hospitalization, and death in just a few minutes. This is complemented with comprehensive inputs including age, gender, health conditions, active cases, vaccination (type and dose), use of any face mask, and the number of people in close contact outdoors or indoors.

We make sure to consider all the important factors when evaluating various risks associated with COVID-19. However, due to the inherent uncertainty in accurately estimating risks, we provide a range of potential risks rather than a single value. Additionally, we assume that individuals that the user encounters are unvaccinated and not wearing masks, as we are unsure about the extent of COVID-19 precautions in a given area. This assumption could slightly inflate the risk in situations where there is high adherence to mask-wearing and vaccination. In future risk assessments for private gatherings or venues with proof of vaccination requirements, we suggest incorporating the behavior of others. We are also interested in incorporating users’ and their peers’ immunity from prior infections. Currently, our calculations do not account for the impact of waning immunity from vaccines or natural infections or how variants of concern may affect the rate of decline. We plan to include these factors in our next release. Moreover, we have not factored in the risk of myocarditis associated with vaccination or infection, a concern that many people have asked us to address. Incorporating this information may demonstrate that the risk of myocarditis is higher with SARS-CoV-2 infection than with vaccination.

While there are areas for improvement, our risk calculator has a wide range of features. Through studies conducted with the risk calculation system, we have found that there are significant synergies between the use of masks with different fitted filtration efficacy (FFE) and different dosages of vaccination, resulting in a decrease in risk. Our risk calculator takes into account all significant variants currently known, including alpha, beta, gamma, delta, and omicron, as well as the increase in risks associated with the presence of a particular variant in a particular region/country. The system updates regularly to fetch the latest number of confirmed cases and variants for 203 countries, making it a reusable tool for the future. The risk calculator has a minimalistic design and can be translated into a web application, making it highly accessible and usable even with slow internet connections. These features make the system highly scalable for users worldwide.

**Conclusion:** The CovARC COVID-19 risk calculator is a valuable and accurate tool that can assist the public and policymakers in assessing the range of risks of infection, hospitalization, and death from COVID-19. The calculator is more comprehensive and simpler than existing alternatives, with a streamlined interface that allows users to obtain risk estimates quickly. The calculator takes into account all significant factors, including age, gender, health conditions, vaccination status, use of face masks, and the number of people in close contact. Although there is always uncertainty in risk estimation, the calculator provides a range of risk estimates rather than an absolute value. We further plan to improve the calculator by including factors such as previous infection, immunity, waning immunity, and the risk of myocarditis with vaccination or infection. The calculator is regularly updated to include the latest information on variants and confirmed cases for over 203 countries, making it a reusable and highly scalable tool. We hope that this calculator will empower the public to live their lives safely and that a beneficial added effect of using the calculator will be education about risk reduction, which ultimately could result in reduced COVID-19 cases. Future directions will include quantifying the impact of CovARC on COVID-19 community transmission levels. Gamifying risk reduction could be a valuable public health strategy for this and future Pandemics.

## Methods

### Dataset usage

The risk calculation system uses a variety of different datasets that are extracted from numerous online sources. Our tool carries out a 14-days aggregate in order to estimate the number of active cases. Therefore, the confirmed cases dataset is updated on a daily basis. As for the variants dataset, we take the value from 31 days ago due to the prevalence of a variant for 1 month in the population. We initially carry out pre-processing of the number of confirmed cases obtained through the Johns Hopkins dataset^24^. We then identify the number of active cases by subtracting the confirmed cases for a given day from the confirmed cases for the previous day and compute a 14-days aggregate for the number of active cases. We carry out a 14-days aggregate due to the fact that the SARS-CoV-2 virus is active for 14 days in any specific condition^33^. We determine a range of 14-days aggregate active cases by taking the upper limit as the product of the 14-days aggregate active cases and the ratio between the confirmed cases obtained from Facebook’s COVID-19 Trends and Impact Survey^34^ and Johns Hopkins dataset^24^. Following this, we extract the variants data using the CDC variants dataset^27^ for calculating the number of different variant cases present in a particular region/country. We estimate the prevalence of a particular variant by carrying out Gaussian smoothing^35^ taking the variant values 30 days before the current date to account for uneven and delayed data reporting^36^.

**Table 2:** A. The first table represents fitted filtration efficacy (FFE)^29,37,38^ for different types of masks currently available on the market. These values are used in order to calculate the reduction in different risks associated with COVID-19 when different types of masks are used. B. The second table represents the efficacy^39–48^ of different types of vaccines against COVID-19 and its variants. This table is used in order to identify reduction is risks associated with COVID-19 when different types of vaccines and dosage is taken.

A. | Mask type	FFE					
2-layer woven nylon mask without nose bridge	0.447					
2-layer woven nylon mask with nose bridge	0.563					
2-layer woven nylon with nose bridge and filter insert	0.744					
2-layer woven nylon with nose bridge washed	0.79					
Cotton Bandana folded surgeon general style	0.49					
Cotton Bandana folded bandit style	0.49					
Single-layer woven polyester gaiter	0.378					
Single-layer woven polyester mask with ties	0.393					
Non-woven polypropylene mask with fixed ear loops	0.286					
3-layer knitted cotton mask with ear loops	0.265					
N95 respirator	0.984					
Surgical mask with ties	0.715					
Procedure mask with ear loops	0.385					
Procedure mask with loops tied, corners tucked	0.603					
Procedure mask with loops tied, corners tucked and ear guard	0.617					
Procedure mask with Clawed hair clip	0.648					
Procedure mask with fix-the-mask technique (rubber bands)	0.782					
Procedure mask with Nylon hosiery sleeve	0.802					
No Mask	0					

B. | Vaccine	Normal	Alpha	Beta	Gamma	Delta	Omicron
Pfizer (Dose 1)	0.8–0.91	0.49	0.36–0.375	0.36–0.37	0.33	–
Pfizer (Dose 2)	0.95	0.87–0.95	0.72–0.85	0.75–0.77	0.79–0.92	0.07–0.1
Pfizer (Dose 2) + Pfizer Booster	0.95	0.87–0.95	0.72–0.85	0.75–0.77	0.79–0.92	0.44-0.47
Pfizer (Dose 2) + Moderna (Booster)	0.95	0.87-0.95	0.72–0.85	0.75–0.77	0.79–0.92	0.63–0.66
Moderna (Dose 1)	0.8–0.9	0.49	0.72	0.72	0.33	–
Moderna (Dose 2)	0.9–0.96	0.91–0.96	0.9–0.96	0.9–0.96	0.855–0.96	0.35–0.52
J &J (Dose 1)	0.69–0.77	0.77	0.52–0.57	0.51–0.68	0.49–0.78	–
J &J (Dose 1) + J &J Booster	0.69–0.77	0.77	0.52–0.57	0.51–0.68	0.49–0.78	0.85
Astrazeneca (Dose 1)	0.55–0.67	0.33–0.37	0.1–0.11	0.11–0.243	0.329	–
Astrazeneca (Dose 2)	0.82–0.85	0.66–0.74	0.22–0.49	0.22–0.49	0.59	–
Astrazeneca (Dose 2) + Pfizer/Moderna (Booster)	0.82–0.85	0.66–0.74	0.22–0.7	0.22–0.49	0.59	0.59–0.62
Novavax (Dose 1)	0.904	0.863	0.486	–	–	–
No Vaccine	0	0	0	0	0	0

**Table 3:** The given table explains the influence of Age group^50^, presence of a specific variant^51^, gender of the user as well as past chronic illness/health conditions (Diabetes, Heart Disease, Cancer, Lung disease, High Blood Pressure, Immunocompromised, Asthma, Kidney Disease, Obesity, Sickle Cell Anemia, HIV, Liver Disease) on risk of hospitalization and death^52^. These factors are taken into account in our risk calculator when we calculate the risk of hospitalization and risk of death from the risk of infection for an individual^53^.

	Hospitalization rate (%)	Death rate (%)
By age group		
0–17 years old	0.8%	0.0015%
18–49 years old	2.5%	0.07%
50–64 years old	7.9%	0.7%
65+ years old	23%	6%
All ages	5%	0.75%
By SARS-CoV-2 Variant—fold higher risk compared to original variants		
Alpha (B.1.1.7, B.1.1.7 with E484K^49^)	1.5(1.5–1.6)	1.6(1.4–1.7)
Beta (B.1.351)	Under Investigation	Possibly Increased
Gamma (P.1)	Possibly Increased	1.5 (1.2–1.9)
Delta (B.1.617.2)	2.3 (1.9–3.0)	2.4 (1.5–3.3)
By Gender—Fold Higher Risk		
Male	–	1.5–2.3
Female	–	1
By any chronic health condition—fold higher risk	2.5	1.2–6.9

In addition to the variants and confirmed cases datasets that we obtain from online sources, we create custom datasets where we used information provided by several different research studies and articles. These datasets consiss o f the mask’s fitted filtration efficacy (FFE) dataset (see Table 2 A) and the efficacy of the vaccine against different variants of virus dataset (see Table 2 B) which we use in order to estimate risk reduction. The vaccine efficacy dataset is coupled with different variants that are extracted using the GISAID dataset to estimate the overall impact of variants with a specific level of vaccination or no vaccination. The mask dataset is used to estimate the overall reduction in risk.

In order to add the influence of indoor and outdoor environment, age, gender, past chronic illness, and variants on risk of infection, hospitalization and death, we further add a multiplier which helps us to accurate the risks associated with carrying out a specific activity during the COVID-19 pandemic. We use all of these datasets and consider the number of people the user passes outdoors while traveling to the location and the number of people they are with at the destination (which can be indoors or outdoors) to estimate several risks associated with COVID-19 (see Table 3).

### Pipeline and workflow

As shown in Fig. 2, we then identify the new cases from the confirmed cases by subtracting the confirmed cases for a given day from the confirmed cases for the previous day. We then identify the number of new cases by taking a 14-days sum for the number of active cases($n_{ac}$). Upon providing the input for region/state within the country and present-day date, we identify the 14-days sum of the active case for a particular previous day and region/state. We then consider the region and date to identify the variants and the average number of variant cases over 30 days. We use the ratio between the confirmed cases reported through Facebook surveys^34^ and the officially reported confirmed cases and multiply them with the confirmed cases reported by Johns Hopkins University dataset^24^ in order to estimate the upper limit for the number of reported cases.

We calculated the reduced risk of infection for variants of SARS-CoV-2 by subtracting one from the efficacy of vaccines and fitted filtration efficacy of masks using the same method. For vaccines, we use the lower and upper limits of efficacy to estimate and multiply it with the higher and lower limits of confirmed cases, respectively and estimate the risk of infection. We then calculate the risk of variants by considering the regional data to identify the number of variants present and multiply them with reduced risk after vaccination to identify the added risk of variants ($r_{v}$). Furthermore, we multiply the number of people passed by indoors ($n_{i}$), outdoors ($n_{o}$), risk due to active cases per unit population ($r_{ac}$), risk reduction due to vaccination ($r_{v}$) and reduced risk due to mask ($r_{m}$) to calculate indoor ($r_{i}$) and outdoor risk ($r_{o}$) range of infection. The sum of this helps us to estimate the range of cumulative risk of infection.

We further estimate the risk of hospitalization ($r_{h}$) and risk of death ($r_{d}$) by using factors related to age ($f_{a}$ and $f_{a1}$), gender($f_{g}$ and $f_{g1}$), past chronic illness($f_{c}$ and $f_{c1}$) and type of variants ($f_{v}$ and $f_{v1}$) and multiply the range of risk factors with the upper and lower limits of the cumulative risk of infection. Finally, we use it to compute the range of risk of hospitalization and risk of death.

### Testing and deployment of web application

Upon finishing the implementation of the risk calculation system as well as several studies related to its robustness, we proceeded to create a web application that could enable the risk calculation system to be user-facing. We used shinyapps^32^ to translate the R code into a web application by creating a simple form-like user interface. Using Rshiny also enabled us to ensure that the webpage is lightweight and can be used with a slow internet connection as well. With this tool, we provided a channel where anyone could access the risk estimation system to identify the personal risk of infection, hospitalization, and death when carrying out daily activities. In order to make it more accessible, the web page’s user interface was also translated for smartphones. This is useful to increase the accessibility of the risk calculator web application to the general public and enable smooth implementation of the system in the daily life of individuals. The representation of the webpage can be seen Fig. 3.

### Supplementary Information

Supplementary Information.

## Data Availability

All of the data used in this study is publicly available and can be either directly accessed or accessed upon filing a request. The data related to the number of active, confirmed, and death cases due to COVID-19 is maintained by the Center for Systems Science and Engineering (CSSE) at Johns Hopkins University at https://github.com/CSSEGISandData/COVID-19. The data for the SARS-CoV-2 variants can be obtained upon request from the repository maintained by Global Initiative on Sharing Avian Influenza Data (GISAID) Initiative https://gisaid.org/hcov19-variants. Facebook/Meta COVID-19 Data used for cross-validation of the number of confirmed cases can be accessed at https://dataforgood.facebook.com/dfg/tools/covid-19-trends-and-impact-survey. Factors used for calculating risk reduction by use of masks and different dosages of vaccines can be seen in Table 2A and Table 2B. Factors used for calculating the risk of hospitalization and death can be seen in Table 3. The source code that supports the findings of this research is available from the corresponding author upon request.
