# Supplementary material for: COVID-19 activity risk calculator as a gamified public health intervention tool
Source: Sci Rep. 2023 Aug 11;13:13056. doi: 10.1038/s41598-023-40338-8 (PMC10421890; doi:10.1038/s41598-023-40338-8)
Supplement: Supplementary file 1 — Supplementary Information. [file 41598_2023_40338_MOESM1_ESM.pdf]

Supplementary Materials

Risk of infection in the case of Delhi, India

Our main focus in carrying out this study was to create an easy-to-use risk calculator that could be implemented for a large set of countries with the least amount of internet bandwidth needed. To test out whether the data we extracted as well as the workflow we followed was effective in accurately estimating risks associated with carrying out a daily life activity for an individual. We carried out a secondary test taking the example of Delhi, India.

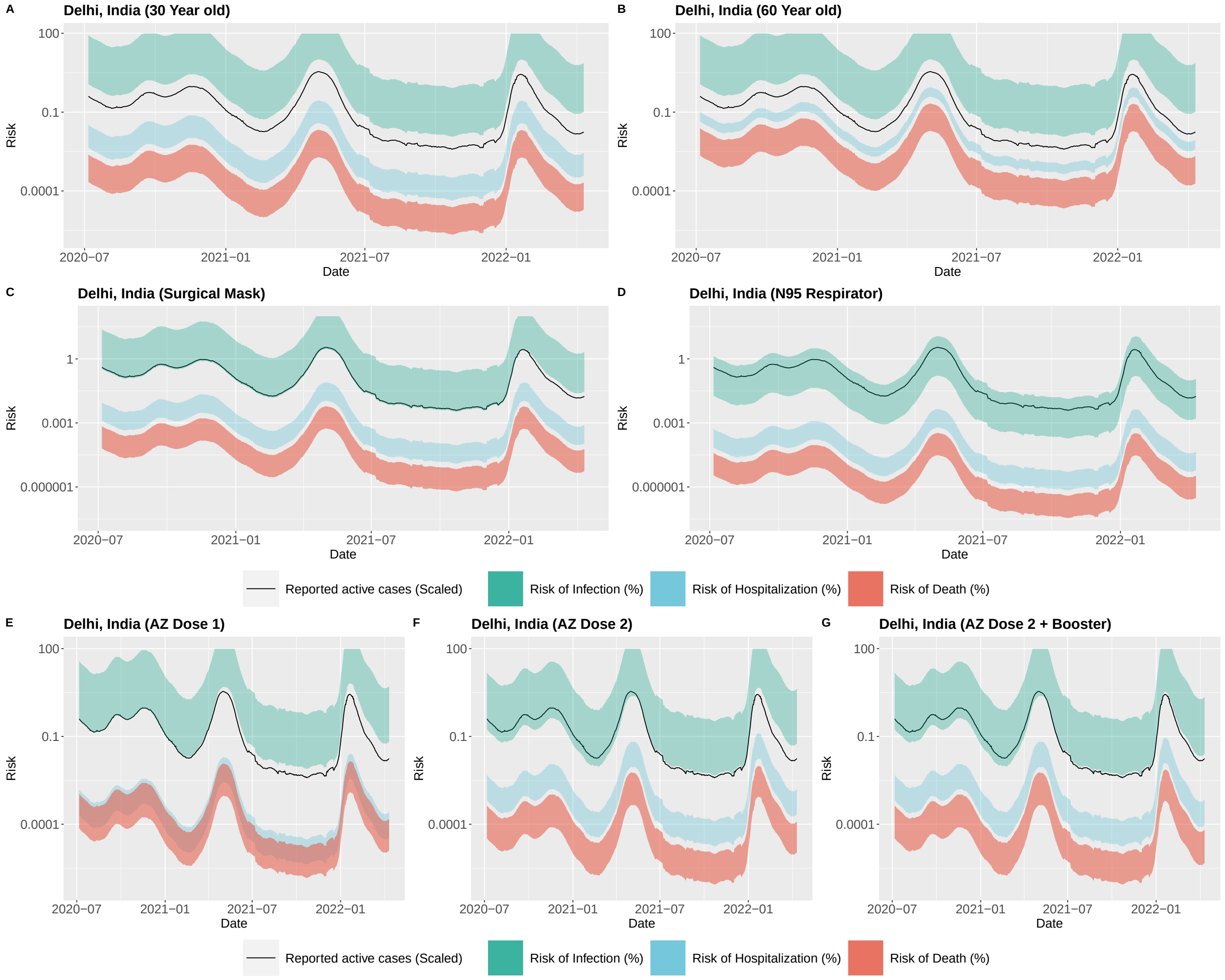

Figure S1. In the following figures, we use the location of Delhi, India and calculated the ranges of risk of infection, hospitalization and death for a **A.** 30-year-old male with no chronic illness, no mask and no vaccination, 10 people passed outdoors and 5 people passed indoors during the activity, a **B.** 60-year-old male with no chronic illness, no mask and no vaccination, 10 people passed outdoors and 5 people passed indoors during the activity. a **C.** 30-year-old male with no chronic illness, surgical mask and no vaccination when 10 people are passed outdoors and 5 people passed indoors during the activity, a **D.** 30-year-old male with no chronic illness, N95 respirator mask and no vaccination when 10 people are passed outdoors and 5 people passed indoors during the activity, a **E.** 30 year old male with no chronic illness, no mask and Dose 1 of AstraZeneca vaccination when 10 people are passed outdoors and 5 people indoors during the activity, a **F.** 30-year-old male with no chronic illness, no mask and Dose 2 of AstraZeneca vaccination when 10 people are passed outdoors and 5 people indoors during the activity and a **G.** 30-year-old male with no chronic illness, no mask and Dose 2 with a booster dose of AstraZeneca vaccination when 10 people are passed outdoors and 5 people indoors during the activity.

In order to further validate the robustness of our risk calculator in the estimation of risk associated with COVID-19 in different countries, we carried out a secondary analysis where we used the location of Delhi, India, in order to estimate different risks associated with COVID-19 when carrying out a daily life activity. Using our risk calculator, we performed a test similar to the previously performed risk estimation for Franklin, MA, USA but for the case of Delhi, India. We did not have county-level

information therefore, we carried out the test on a city level. We first calculated the range of risk of infection, hospitalization, and death with change time for a 30-year-old and 70-year-old male living in Delhi, India, with no chronic illness, no vaccination, no mask, five people passed indoors, and ten people passed outdoors (see Supplementary Figure S1 A., B.). We then conducted the same test but only took the case of a 30-year-old male when AstraZeneca vaccination first, second and booster doses were taken in order to check the reduced range of risk of infection. (see Supplementary Figure S1 E., F., G.). We then also carried out the tests related to the reduction in range of risk of infection when different types of masks are used (see Supplementary Figure S1 C., D.).

As we can see with our results, we were able to observe an increase in the risk of hospitalization and risk of death with an increase in the age of the user. Furthermore, there is a high risk of hospitalization and death during the second wave (when the delta variant was observed) compared to the third wave (when the omicron variant was observed). There is also a reduction in risk of infection when different doses of vaccination are taken by the user which is similar to what we observed in the case of Franklin, MA, USA. Furthermore, the reduction in risk is also very high when different types of masks are used by the user which is analogous to the results that we previously obtained.

Calculation of Risk for different Countries

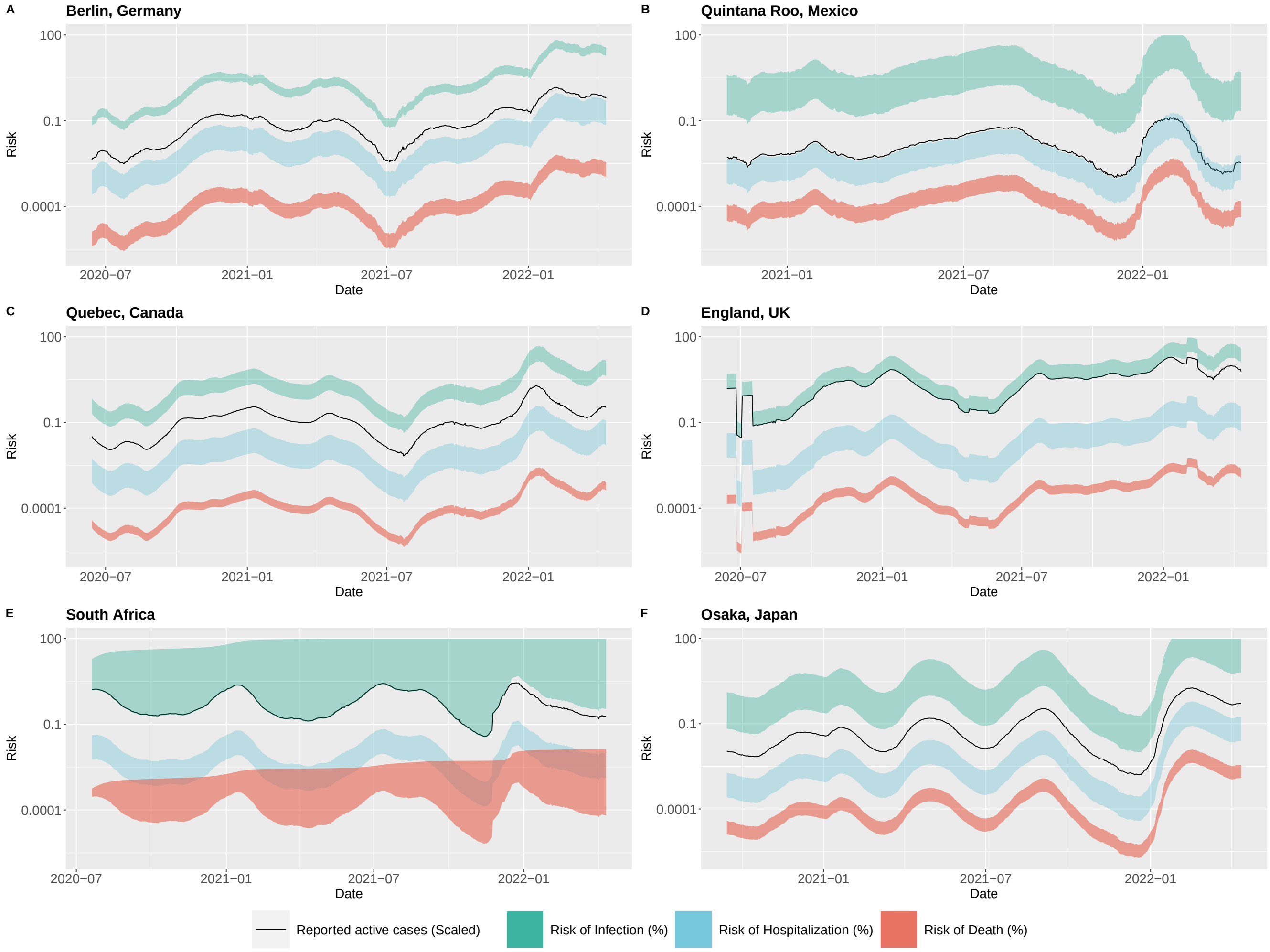

Figure S2. In the given figure, we estimated the ranges of risk of infection, hospitalization, and death for a 30-year-old male with no past chronic illness, no mask, and no vaccination carrying out an activity involving interaction with 10 people outdoors and 5 people indoors in the locations **A.** Berlin (Germany), **B.** Quintana Roo (Mexico), **C.** Quebec (Canada), **D.** England, United Kingdom, **E.** South Africa and **F.** Osaka, Japan

We further tested out the risk calculator over the data for 6 other regions and countries. The regions included Berlin (Germany), Quintana Roo (Mexico), Quebec (Canada), England (United Kingdom), Osaka (Japan) and South Africa. Since South Africa did not provide regional-level information, we estimated the risk on a country level (see Supplementary Figure S2 A., B., C.,

D., E., F.).

For each of the regions, we estimated the risk by taking an example of a 30-year-old male who is going to carry out an activity involving interaction with 10 people outdoors and 5 people indoors, with no past chronic illness, no masks and no vaccinations. The results were consistent with our previous findings. The risk scores varied across regions, with some regions experiencing higher risk levels than others. For instance, Berlin (Germany) had a relatively low-risk score compared to South Africa or Quintana Roo (Mexico), which had a significantly higher risk score. Overall, the results demonstrated the effectiveness of the COVID-19 risk calculator in assessing the risk levels of different regions and countries.

Using this test we were able to clearly check the validity of our risk calculator for different regions and countries across the world. It helped us in ensuring that the calculator is useful for estimating several individual-level risks related to carrying out any specific activity during COVID-19 for 203 countries across the world whose data we also had available.

Calculation of Risk for different Age Groups

The next test that we wanted to perform was to estimate the change in the risk of infection, hospitalization and death across different age groups. For this case, we used the example of England (United Kingdom) and estimated change in the trend of risk of infection, hospitalization and death for a 5-year-old, 21-year-old, 55-year-old and 70-year-old male with no past chronic illness, no mask and no vaccination performing an activity involving interaction with 10 people outdoors and 5 people indoors (see Supplementary Figure S3 A., B., C., D.).

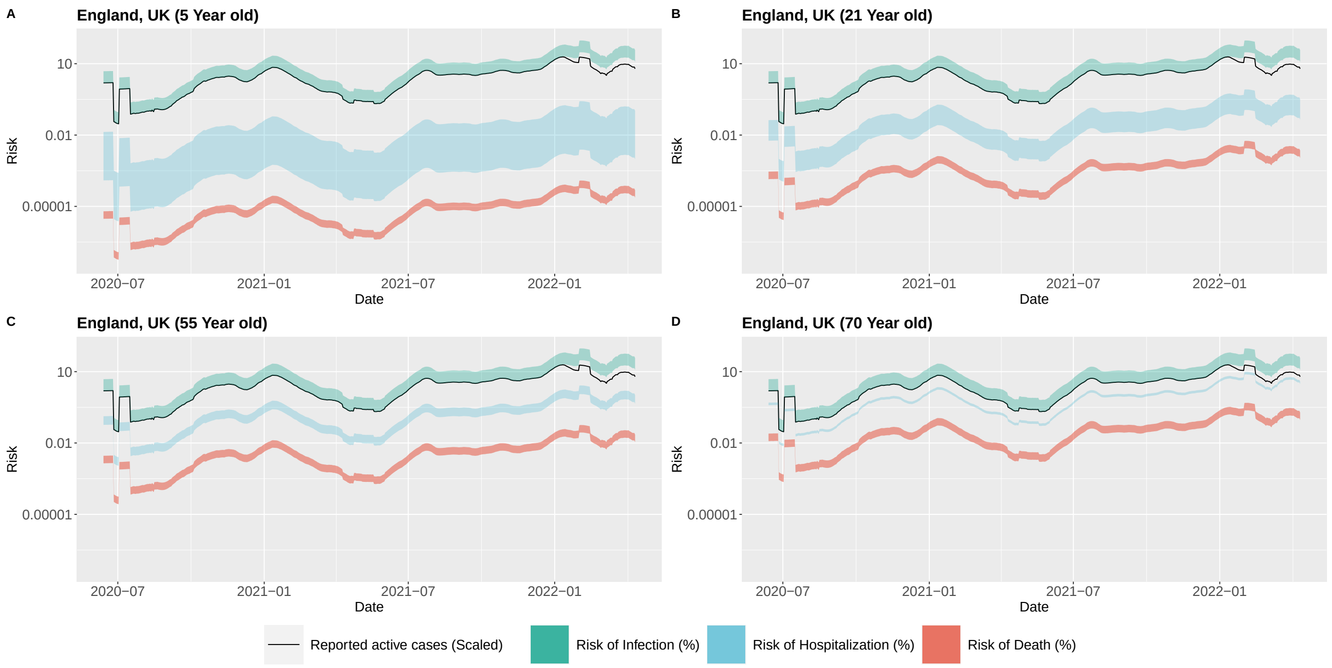

Figure S3. In the given figure, we estimated the ranges of risk of infection, hospitalization, and death for a male with no past chronic illness, no mask, and no vaccination carrying out an activity involving interaction with 10 people outdoors and 5 people indoors in the location of England (United Kingdom) at the age of **A.** 5 years, **B.** 21 years (M, **C.** 55 years and **D.** 71 years.

We found that the risk of infection, hospitalization, and death varies significantly across age groups, with older individuals having a higher risk compared to younger individuals. For instance, for the example scenario in England, the risk of infection was found to be the highest for the 70-year-old male, followed by the 55-year-old, 21-year-old, and 5-year-old males, in that order. Similarly, the risk of hospitalization and death also increased with age. These findings highlight the importance of age as a significant factor in assessing COVID-19 risk and the need for age-specific risk assessment tools. This inspired us to include age-specific risk estimates in future version updates of CovARC to further improve its accuracy and usefulness.

Simulating different scenarios for variants

As the next step, we wanted to simulate different scenarios where there was the presence of either no variant or one specific variant to check the change in the trend of ranges of risk of infection when a specific dosage of vaccination was taken and when no vaccination was taken. In order to do so, we created 6 scenarios wherein there was the presence of no variant, only alpha

variant, only beta variant, only gamma variant, only delta variant and only omicron variant. We then simulated a scenario where there was Dose 1 of Pfizer vaccination taken for the case of no variant presence and only alpha or beta or gamma variant presence. Furthermore, we simulated another case where Dose 2 of the Pfizer vaccination was taken in the presence of only the delta variant and a last case where Dose 2 and a booster dose of the Pfizer vaccination were taken in the presence of only the Omicron variant. The test was performed for a 30-year-old male living in Franklin, Massachusetts, USA with no past chronic illness and mask carrying out an activity involving interaction with 10 people outdoors and 5 people indoors (see Supplementary Figure S4 A., B., C., D., E., F.).

In addition to the effectiveness of different vaccine doses for different variants, the simulation also allowed us to observe the impact of variant presence on the ranges of risk of infection. The results demonstrated that the range of risk of infection was significantly higher in the presence of the delta and omicron variants compared to the other variants and no variant scenario. This highlights the importance of continued monitoring and research on the impact of emerging variants on COVID-19 transmission and the efficacy of vaccines. The simulation also provided valuable insights into the potential benefits of booster doses for the omicron variants, which could inform future vaccination strategies. Overall, the results of this simulation emphasize the importance of taking into account the presence of variants when evaluating the effectiveness of vaccines and assessing the ranges of risk of infection.

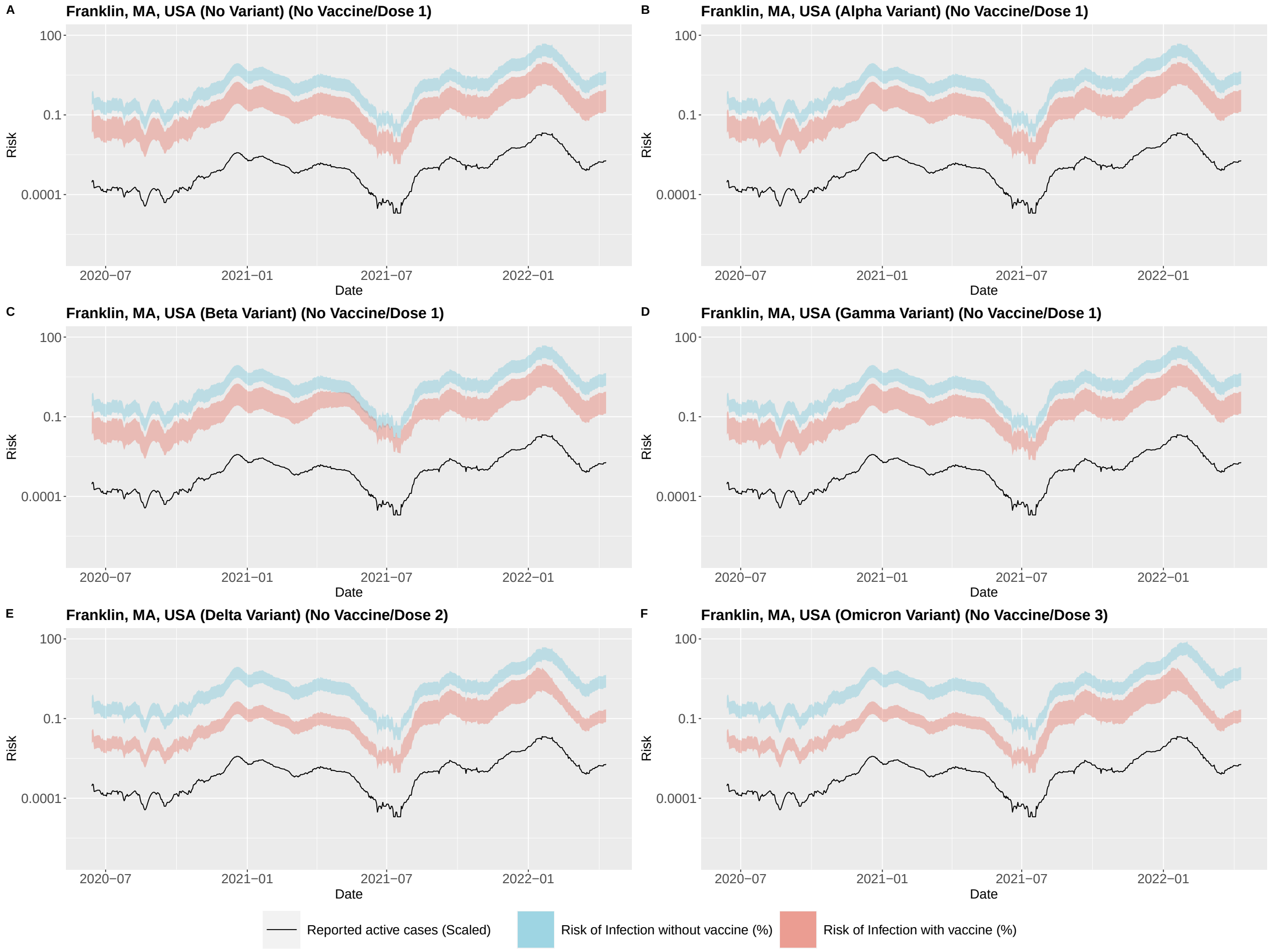

Figure S4. The given figure illustrates the ranges of risk of infection for a simulated scenario of a 30-year-old male with no past chronic illness and no mask vaccination carrying out an activity in Franklin, Massachusetts, USA involving interaction with 10 people outdoors and 5 people indoors when there is the presence of **A.** No variants with no vaccination and no variants with Dose 1 of Pfizer vaccination, **B.** Only Alpha variant with no vaccination and only Alpha variant with Dose 1 of Pfizer Vaccination, **C.** Only Beta variant and no vaccination and only Beta variant with Dose 1 of Pfizer Vaccination, **D.** Only Gamma variant with no vaccination and only Gamma variant with Dose 1 of Pfizer Vaccination, **E.** Only Delta variant and no vaccination and only Delta variant with Dose 2 of Pfizer Vaccination, **E.** Only Omicron variant and no vaccination and only Omicron variant with Dose 2 & Booster dose of Pfizer Vaccination
